# SPI-RO-ME-*TROL*-O-*GY*

**Published:** 1854-03

**Authors:** 


### HALL'S JOURNAL OF HEALTH.

VOL. I.] MARCH, 1854. [NO. III.

### SPI-RO-ME-*TROL*-O-*GY*,

PRONOUNCED with the accent on the ante-penult, orfourth syllable, teaches the measurement of the breath, and, by a little license, the lungs themselves, as the breath is contained in the lungs. If a man has all his lungs within him, in full operation, it is impossible for him to have consumption, whatever may be his symptoms, because consumption is a destruction of a portion of the lungs, and when that is the case they can no more have the full amount of breath or air than a gallon measure can hold a gallon after its size has been diminished by having a portion of the top off or removed.

It becomes, then, of great importance to accomplish two things:—

First, to measure accurately, and with as much certainty as you would measure wheat by a standard and authentic bushel measure, the amount of air contained in the lungs.

Second, to ascertain what amount of air the lungs ought to contain in full and perfect health.

The chemist has no difficulty in measuring out to you a cubic foot of gas. The gas which lights our dwellings and which burns in the streets of cities, when the moon don't shine, is *capable* of being accurately measured, and so is the air we breathe, with equal simplicity and certainty, even to the fraction of a cubic inch.

Take a common tub or barrel, of any height, say two feet, and fill it with water; get a tin cup of equal length, and of such a circumference that each inch in length should contain ten cubic inches of air or water, turn this tin cup bottom upward in the barrel of water, make a hole in the bottom of the tin cup, insert a quill or other tube into this hole, take a full breath, and then blow out all the breath you can at a single expiration through this quill; the air thus expired gets between the surface of the water and the bottom of the tin cup, and causes the tin cup to rise; if it rises an inch then you have emptied from your lungs into the cup ten cubic inches of air; if you cause the cup to rise twenty inches, then your lungs have measured out two hundred cubic inches of air, and by dividing the cup into tenths of inches, you will be able to ascertain the contents of the lungs to a single cubic inch.

This is a lung measure of the simplest form; it must be so arranged with a pulley on each side of the cup, each pulley having a weight of half the weight of the cup, so as to steady the cup when it rises, and keep it at any point, as lamps are sometimes suspended in public buildings.

Being able then to measure the amount of air the lungs do hold, down to an inch or even a fraction of an inch if desired, the next point to know is how much air ought a man's lungs contain when he is in perfect health; for if a man in sound health can expire or measure out two hundred cubic inches of air, it is easy to see that if his lungs are half gone he can give out but one hundred cubic inches, and so of any other proportion large or small, and the grand practical conclusion is that when a man can breathe out the full quantity, all his lungs must be within him, and the presence of consumption is an utter impossibility in that man; and even if this was the only point to be learned, what a glorious truth it must be to the man who was apprehensive of his being consumptive, that such a thing is simply an impossibility, demonstrably so by figures and by sight. He can see it for himself without the necessity of leaning doubtfully, *so doubtfully*, sometimes, on the judgment, or expressed opinion of his physician.

To find out how much air a healthy man's lungs should hold, we must act precisely as we would in determining the quantity of anything else; we must experiment, observe, and judge. We have decided long ago on the average weight of men, their average amount of blood, the average weight of the brain; and surely there ought to be some method of determining the average amount of a man's lungs. But this last would not be sufficiently accurate, to make it safely practical; we must be able to say to this man, your lungs, if sound and well, will hold so much; and to another, so much, for the amount of breath is as various as the amount of brain. A large head has a large amount of brain of some kind or other, and so a large chest must have a large quantity of lungs to fill it; these are general truths only. If a man six foot high, and known to be in perfect health, will give out from his lungs at one expiration two hundred and sixty-two cubic inches of air, that is a fact to begin with.

If a thousand healthy six-footers, or ten thousand, do not fail in one single instance to give out as much, then we may conclude that any other man as tall, who gives out as much, is also healthy *as to his lungs*, and at length the facts become so cumulative that we feel safe in saying that any man, six feet high, who can breathe out at one single effort two hundred and sixty-two cubic inches of air, that man must have all his lungs within him, and that they are working fully and well.

But if in pursuing these investigations, in the same manner, as to healthful men five feet high, we observe that in any number of thousands, not one single one ever fails to give one less that one hundred and sixty-six inches, and that any other number of thousands, five feet seven inches high, and in acknowledged perfect health, never fail in one solitary instance to give out two hundred and twenty-two cubic inches of air, then a thinking man begins to surmise that the amount of lungs a man in health has, bears some proportion to his height; this is found to be the actual fact of the case. And without being tedious I will give the result, that for every inch that a man is taller, above a certain height, he gives out eight more cubic inches of air, if he is in sound health, as to his lungs.

Let the reader bear in mind that these are the general principles—circumstances modify them. But I do not want to complicate the subject by stating those modifications at present. I wish the reader first to make one clear simple truth his own, by thinking of it, and talking about it, when occasion offers, for a month—then I may say more.

But, for the sake of making a clear, distinct impression, let us recapitulate:—

1. The amount of air which a man's lungs can expire at one effort can be accurately and uniformly measured, down to the fraction of a cubic inch.
2. The amount of air which a healthy man's lungs hold is ascertained by cumulative observations.

3. That the amount thus contained is proportioned to the man's height.
4. That that proportion is eight cubic inches of air for every additional inch of height above a certain standard.

With these four facts, now admitted as such, inferences may be drawn of great interest in connection with other observations, which any reader who takes the trouble may verify.

Observation 1st.—I have never known a man who was in admitted consumption, and whose subsequent death and *post-mortem* confirmed the fact, capable of measuring his full standard.

Observation 2d.—In numerously repeated instances, persons have been pronounced to have undisputed consumption, and as such were abandoned to die, but on measurement they have reached their full standard, enabling me to say that they had not consumption, and their return to good health, and their continuance in it for years after, and to this day, is an abiding proof of the correctness of my decision.

Observation 3d.—No persons have come under my care, who died of consumption within a year, who, at the time of examination reached their full lung measurement.

Observation 4th.—Therefore, any man who reaches his standard, has reason to believe that he cannot die of consumption within a year, an assurance which, in many cases, may be of exceeding value.

Observation 5th.—As a man with healthy lungs always reaches his full standard, and as it is impossible for a consumptive man to measure his full standard, then it may be safely concluded that a man cannot die of consumption while he gives his healthy measure, and also that he who cannot measure full, is in danger, and should not rest a single day, until he can measure to the full.

When persons are under medical treatment for deficient lung measurement, accompanied with the ordinary symptoms of common consumption, they improve from week to week in proportion as they measure out more and more air from the lungs: on the other hand, when they measure less and less from time to time, they inevitably die. With this view of the case, the reader will perceive that as a general rule a man can tell for himself, as well as his physician, whether he is getting well or not, and, as an illustration, an article is copied verbatim from the eighth edition of "Bronchitis and Kindred Diseases," Redfield publisher, page 361, on

"THE MATHEMATICAL MEASUREMENT OF THE LUNGS AS A SIGN OF CONSUMPTION.

"The lungs contain air; and their object is to receive, hold, and expel air; a certain amount of this air is necessary to the health of any individual, but that amount must vary in proportion to the size and age of a person, as much as the healthful amount of blood is proportionate to the size and age.

"It is known how much air a man's lungs, in perfect and full healthful operation, should hold, by measuring it as we would measure water, by transferring it from a vessel whose capacity was not known into one whose capacity was known. If, then, I find that every man of thousands, who is in perfect health, emits a certain amount of air from his lungs, I conclude that any other man, *under similar circumstances*, who gives from his lungs an equal amount of air, must be in good health, as far as his lungs are concerned, and every year accumulates its additional proofs of the same great fact, and when it is known that the lungs work *fully* and well, an immense burthen is at once removed from the mind of the physician, as well as patient, for he has less to do—the patient has less to dread.

"All that the *Spirometer* does, (or *Breath-Measurer*, which is its literal signification,) is to measure the amount of air contained in any man's lungs with mathematical certainty and precision, down to the fraction of a single cubic inch. Thus far the patient can see, as well as the physician, what is his actual measure; and by comparing it with what it ought to be in health, he can have some idea of what he has to do, and of his present condition.

"We all must know that if a man's lungs in health should hold three hundred cubic inches, they would, if half gone, certainly not measure over one hundred and fifty, *and so of any other proportion*, down to an inch.

"The two important uses to be made of this most invaluable principal are—

*First.* If a man can only expire his full healthful quota of air, he most assuredly cannot have actual consumption, whatever else may be the matter with him, and the knowledge of this one fact alone, arrived at by such unmistakable evidence, is of incomputable worth to any invalid, not only relieving him of the weight of a million mill-stones, but in affording him an important means of restoration—*hopefulness*, for we almost all instinctively feel, if it is not consumption there is at least a chance of life; but if it is consumption there is no hope.

"*Second.* The next important practical deduction is of a two-fold character.

"If the lungs do not give out their full healthful amount of air, it is because they are actually affected or are threatened. The instrument does not tell this, it must be determined by the mature judgment of the experienced physician.

"If the lungs be in a consumptive decay, the pulse and auscultation, with the data already afforded by measurement, will detect this state of things, with a degree of certainty which is most admirable; and this certainty is made doubly sure, if being under treatment a short time, his lungs measure *less* week after week, for then he is certainly dying by inches.

"But it does not follow, because a man does not measure to his full standard, that he is consumptive; it only shows the one thing—that he is defective as to the action and capacity of his lungs; that deficiency may be the result of decay, or debility, or from the lungs being crowded with phlegm or other fluids; if the deficiency is not from decay, proper treatment will diminish that deficiency from week to week, because the treatment invites back the action of the lungs. Thus it is that the gradual *increase* in the capacity of the lungs to hold air, when that capacity, by any cause, has been diminished, is demonstrative of a return towards health.

"On the other hand, as persons are declining, the measurement decreases week by week, until there is scarce breath enough to enable them to cross the room, and soon they step into the grave.

### "A WEIGHTY CONSIDERATION.

"Common consumption comes on by slow degrees, and I have never known a case that was not preceded, for months, by an inability of the lungs to measure their full standard. I consider it wholly impossible for a man to have actual consumption, until he has not been able for months to measure the full amount of air. This deficit in the measurement of the lungs *never fails* to exist in any case of clearly defined consumption, and inasmuch as it *always* precedes consumption, its existence for some months in succession ought to be considered a symptom of consumption in its early stages, and a course of treatment should be adopted which would annihilate that deficit at the earliest possible moment.

"To show how certainly this deficit of lung capacity, or lung action, is removed, when it exists not as an effect of a decay of the lungs, but as an effect of imperfect action, I give here a few cases.

"C. W. F., aged 17, an only son of a wealthy family, was placed under my care May 26, 1852. Thin in flesh, pain in side, sore throat, tightness across the breast, short breath, difficult to fetch a long breath, troublesome running and sniffling of the nose, a weak back, with other indications of a weakly constitution. The measurement of his lungs should have been two hundred and twenty-five cubic inches; their actual capacity was two hundred.

| Date. | | Pulse. | Weight. | Breathing. | Lung Measure. |
|---|---|---|---|---|---|
| "May, 1852, | 26, | 72 | 103 | 16 | 200 |
| June | 2, | 72 | 103 | 16 | 206 |
| | 9, | 72 | 103½ | 16 | 216 |
| | 24, | 72 | 107 | 16 | 238 |
| July | 19, | 88 | 104 | 20 | 216 |
| | 23, | 82 | 103 | 18 | 216 |
| August | 7, | 78 | 105 | 15 | 230 |
| | 24, | 76 | 107½ | 16 | 238 |
| Sept. | 29, | 72 | 111½ | 16 | 250 |
| Nov., 1853, | 8, | 72 | 121½ | 16 | 252 |

"The parents of this case, particularly the mother, visited me at different times, expressing the deepest solicitude, and exhibiting an abiding impression that their child, upon whom so many hopes were hung, was certainly going into a decline, especially as he had grown up rapidly, and was a slim, narrow-breasted child.

"The reader will perceive with what admirable promptness the lungs answered to the means used for their development, in the very first fortnight, and with that increase of action a corresponding increase in flesh, so that in four months, and they embracing the hottest of the year, when most persons lose both flesh and strength, he had gained eight and a half pounds, while the capacity of his lungs for receiving air had increased one fifth, that is, fifty cubic inches, and at the end of a year, when he called as a friend, was still gaining in flesh, and strength, and vigor, with no indication, apparent or covert, of any disease whatever.

"What untold treasure would these parents have given, when their child was first brought to me for examination, to have known that the very next year their son would have been one of the most hearty, healthy, manly-looking young men of his age in New York; and yet there can be no doubt that he would have dwindled away, like a flower prematurely withered, had his case been neglected, in the vain hope of his '*growing out of it!*'

"The reader will notice, that on the 13th of July, every symptom became unfavorable; his weight diminished, his breathing was more rapid, and his lung-measurement declined largely. The reason is, that he left the city in June, and spent some weeks at Newport and Saratoga, with his parents, intermitting all remedial means; but, as soon as he returned to New York, and gave diligent attention to what was required of him, his symptoms began at once to abate, and he steadily improved to his recovery. '*The Springs' have proved the grave of many young people with consumptive symptoms*, and older consumptives generally get worse there. The high feeding, or *get what you can* system of diet at watering-places, fashionable hotels, and boarding-houses, their Lilliputian, one-windowed rooms, from one to 'five pair back,' the midnight clatter along interminable passages, the tardy, or no answer, to bell-call, the lookout from your chamber window over some stable, side-alley, or neighbor's back yard; these, with the coldness, and utter want of sympathy at such places, would soon make a well man sick, and will kill instead of cure the consumptive. They want, instead of these, the free, fresh mountain air, the plain substantial food of the country farm-house, the gallop along the highways, the climbing over the hills by day, and the nightly reunions with family and kindred and friends. And yet the *million stereotype this mistake* against all reason and common sense. Only now and then one is found to choose the better way against troops of remonstrants and opposers, who never had experience, who never think for themselves,—and that is the brave man who gets well, especially when he is determined to do so.

"Some years ago I published a compact octavo of a hundred pages, on 'Throat Ail, Bronchitis and Consumption, their Causes, Symptoms and Cure,' giving various illustrations in both cases, with the treatment adopted, but like pretty much all who publish on their own account, copies enough were not sold to pay for the paper, consequently they are yet to be had, mailed *post-paid* to any part of the United States, for one dollar, sent to the Editor's address."

